# Nail Biting as a Cause of Appendicitis

**DOI:** 10.1155/2020/3930905

**Published:** 2020-04-01

**Authors:** Michael Pagacz, Philip Bao, Juan Carlos Alvarez Moreno, Lydia Howard

**Affiliations:** ^1^A.M. Rywlin, MD Department of Pathology, Mount Sinai Medical Center, Miami Beach, FL, USA; ^2^Department of Surgery, Mount Sinai Medical Center, Miami Beach FL, USA; ^3^Herbert Wertheim College of Medicine, Florida International University, Miami, FL, USA

## Abstract

Ingestion of a foreign body is commonly encountered in clinical practice, but most cause no complications, passing spontaneously through the gastrointestinal tract. However, they can cause obstructive signs and symptoms, and surgical intervention for extraction of the foreign body may be required after identifying its location. We present here the case of a 49-year-old woman who presented to our emergency room with abdominal pain localizing to the right lower quadrant. Evaluation was most consistent with acute appendicitis, and she underwent uncomplicated appendectomy. A keratin nail with *Actinomyces* was identified in her appendix. Foreign bodies in the appendix can cause simple appendicitis, perforation, periappendiceal abscess, and peritonitis. Regardless of etiology, an appendectomy often ends up the primary treatment, but unusual and rare causes are worth noting if only for the clinician to be aware of when evaluating the next patient with abdominal pain and considering treatment options or future prevention. Our case is an example of a rare scenario in which an *Actinomyces*-contaminated human nail lodged in the appendix of a woman eventually resulting in acute appendicitis.

## 1. Introduction

Ingestion of a foreign body is commonly encountered in clinical practice, but most cause no complications, passing spontaneously through the gastrointestinal tract. However, they can cause obstructive signs and symptoms, and surgical intervention for extraction of the foreign body may be required after identifying its location.

We present here the case of a 49-year-old woman who presented to our emergency room with abdominal pain localizing to the right lower quadrant. Evaluation was most consistent with acute appendicitis, and she underwent uncomplicated appendectomy. A keratin nail with *Actinomyces* was identified in her appendix.

## 2. Case Report

A 49-year-old woman with surgical history of bilateral mastectomies for breast cancer and hysterectomy with bilateral salpingo-oophorectomy presented to the emergency room with abdominal pain of five-day duration. At that time, she was undergoing IBRANCE® chemotherapy at another institution for hepatic metastases from her breast cancer. She described her symptoms as being of gradual onset and constant. The pain was dull, at first diffuse and later mainly in the lower abdomen but of increasing intensity. It was nonradiating and without alleviating or exacerbating factors. Physical examination was unremarkable except for right lower quadrant abdominal pain around McBurney's point. There was no rebound tenderness, and she was afebrile. Of note, she was leukopenic (WBC 2.4 × 10^3^/*μ*L, range 4.8‐10.8 × 10^3^/*μ*L) and neutropenic (absolute neutrophils 1.08 × 10^3^/*μ*L, range 1.8‐7.2 × 10^3^/*μ*L). Abdominal computed tomography demonstrated a dilated and inflamed appendix grossly consistent with acute appendicitis. Surgical consultation was obtained, and she underwent a laparoscopic appendectomy the next day with no intraoperative evidence of perforation or gangrene. She was discharged home the same day and was doing well in a follow-up approximately two weeks later.

**Figure 1:**
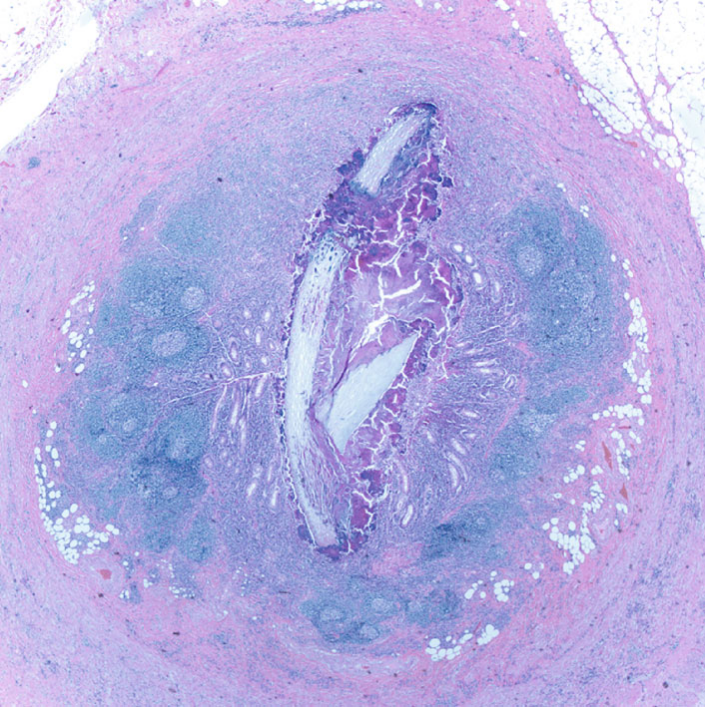
H&E showing a nail in the lumen of the appendix (2x).

**Figure 2:**
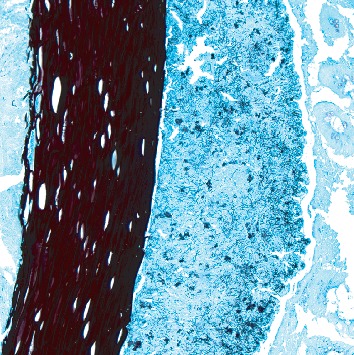
Grocott's methenamine silver stain showing *Actinomyces* species (10x).

**Figure 3:**
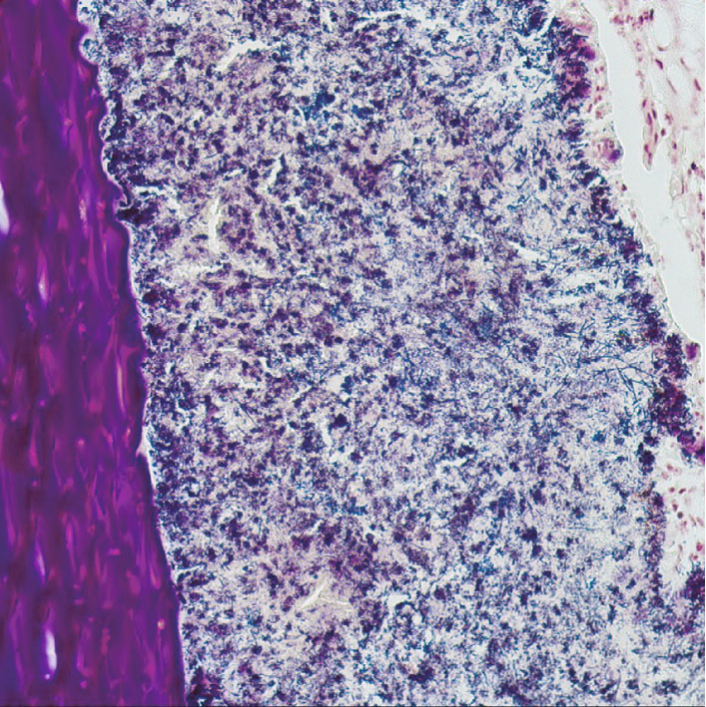
Brown and Brenn stain showing *Actinomyces* species (20x).

Pathology revealed a dilated appendix that measured 6.5 cm in length and 2.6 cm in average diameter. It had a brown and yellow lumen and attached scant fatty tissue. Original hematoxylin/eosin-stained sections revealed a mixed inflammatory infiltrate consisting predominantly of eosinophils and plasma cells. Given a clinical diagnosis of acute appendicitis, deeper sections were examined. They revealed a focally occluded lumen which was filled with foreign material consistent with nail (keratin) encased in organisms with the morphology of *Actinomyces* sp. (Figures 1–3). No fecalith or neoplastic mass was otherwise seen.

## 3. Discussion

Accidental and intentional swallowing of foreign bodies is frequently encountered in clinical practice. This happens more commonly among children, but anyone can be affected. Most foreign bodies transit through the gastrointestinal system and are passed with the stools without the need of surgical intervention [1]. However, bowel perforation or obstruction can occur depending on the object's size and shape. Classically, adult appendicitis is felt due to obstruction of the appendiceal lumen by a fecalith, resulting in ischemia, tissue damage, and subsequent infection. One can thus imagine a foreign body causing the same pathophysiology. However, identifying a foreign body in the appendiceal lumen is extremely rare [2], being reported in approximately 0.005% of the cases of appendicitis [3]. This occurs when the foreign body is heavier than the intestinal bolus causing it to slow down and halt in the cecum during transit at which time it may enter the appendiceal lumen. This occurrence depends on the position of the appendix and whether its orifice is obliterated, small, or wide open. If the appendix is retrocecal, the probability of an object entering into the lumen is very low [4]. Once in the appendix, the object may not be able to reenter the colon due to a lack of peristaltic action and may cause appendicitis and/or perforation. Sharp and pointed objects are more likely to cause perforations and periappendiceal abscesses, which present more rapidly after ingestion. Blunt objects can also cause bowel perforations most likely through slow pressure necrosis [5], are more likely to remain dormant for longer periods, and cause appendicitis by obstructing the appendiceal lumen. Various materials such as needles and other metal objects, as well as organic matter such as seeds, have been implicated as causes of acute appendicitis. Clinical presentation can vary from hours to years. As far as we know, appendicitis caused by a human nail has not been previously described.

Rare cases in which a foreign body in the appendix has not generated inflammation have been described in the literature. In a prospective study involving 62 Eskimo patients with a lead shot pellet in the appendix, none had developed clinical evidence of appendicitis. Barium studies showed no evidence of luminal obstruction of their appendices, and in eight cases where the appendix had been removed, a normal appendix negative for inflammation was found [6]. A case was also reported in which 27 lead shot pellets were found in the appendix of a 45-year-old patient who had eaten pigeons in childhood with no clinical symptoms of appendicitis. It is therefore possible, although rare, that a foreign body in the appendix even after many years may not lead to appendicitis either histologically or clinically [7].

*Actinomyces* is gram-positive facultative anaerobic bacteria found in soil as well as colonizing human gut flora. It can cause opportunistic infections, but actinomycosis is generally rare with a reported incidence between 1/300,000 and 1/1,000,000 [8]. The pathogenesis of abdominal actinomycosis is poorly understood, although disruption of the mucosal barrier is considered a critical step in the process. The appendix and ileocecal region appear to be most commonly involved, and predisposing factors of abdominal actinomycosis include recent abdominal surgery, bowel perforation, neoplasia, poor oral hygiene, and intrauterine contraceptive devices [9]. With respect to appendicitis, *Actinomyces* is reported as the etiology in only 0.02%–0.06% of cases [10]. It has been observed in appendiceal specimens taken from patients with a more subacute clinical presentation as opposed to the progression of classic appendicitis over 12 to 24 hours, and if the diagnosis was known before surgery, treatment with a prolonged course of antibiotics like penicillin alone would likely be sufficient [8].

Our patient was indeed immunosuppressed from chemotherapy treatment and malignancy and thus at risk of actinomycosis opportunistic infection including appendicitis, but more likely it was the foreign body nail which served as the inciting event, entering her appendiceal lumen to cause obstruction and injury. Actinomycosis frequently develops in the nail bed of the hands of gardeners [11]. It is plausible that our patient is a nail biter gardener or she ingested a contaminated nail in prepared food. The *Actinomyces* found in her appendix specimen was thus environmental and introduced with the nail and less likely a result of secondary colonization from her own gut flora. Eventually progressive inflammation and infection resulted in her clinical presentation treated as acute appendicitis. Cases of *Actinomyces* associated with a foreign material in the bronchus exist and are thought due to aspiration from the mouth or oropharynx [12]. If we were able to identify colonizing *Actinomyces* from her mouth or gut flora, genetic comparison with the bacteria from the appendix specimen might ultimately reveal the source.

## 4. Conclusion

Foreign bodies in the appendix can cause simple appendicitis, perforation, periappendiceal abscess, and peritonitis. Regardless of etiology, an appendectomy often ends up the primary treatment, but unusual and rare causes are worth noting if only for the clinician to be aware of when evaluating the next patient with abdominal pain and considering treatment options or future prevention. Our case is an example of a rare scenario in which an *Actinomyces*-contaminated human nail lodged in the appendix of a woman eventually resulting in acute appendicitis.
